# Supplementary material for: Effects of different vegetable rotations on the rhizosphere bacterial community and tomato growth in a continuous tomato cropping substrate
Source: PLoS One. 2021 Sep 23;16(9):e0257432. doi: 10.1371/journal.pone.0257432 (PMC8459948; doi:10.1371/journal.pone.0257432)
Supplement: S1 Fig — CK: Continuous tomato cropping; Q: Celery/tomato rotation; B: Cabbage/tomato rotation; D: Kidney bean/tomato rotation. Different lowercase letters at each phenological stage indicate that the differences are statistically significant (P < 0.05). (DOCX) [file pone.0257432.s001.docx]

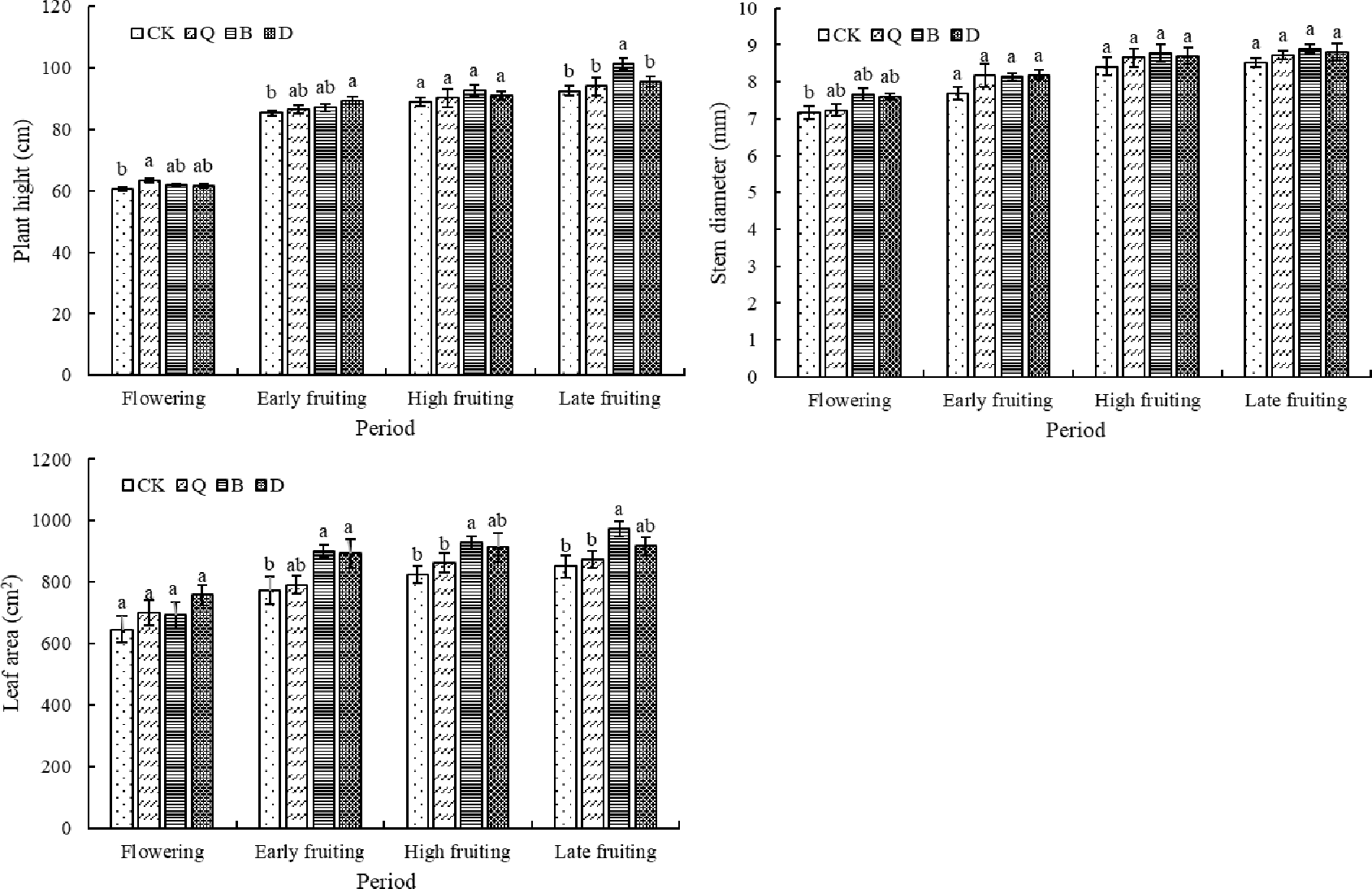


**S2 Figure. Effects of different vegetable rotations on tomato plant growth.CK: continuous tomato cropping; Q: celery/tomato rotation; B: cabbage/tomato rotation; D: kidney bean/tomato rotation. Different lowercase letters at each phenological stage indicate that the differences are statistically significant (P < 0.05).**
