# Supplementary material for: Effects of different vegetable rotations on the rhizosphere bacterial community and tomato growth in a continuous tomato cropping substrate
Source: PLoS One. 2021 Sep 23;16(9):e0257432. doi: 10.1371/journal.pone.0257432 (PMC8459948; doi:10.1371/journal.pone.0257432)
Supplement: S1 Table — CK: Continuous tomato cropping; Q: Celery/tomato rotation; B: Cabbage/tomato rotation; D: Kidney bean/tomato rotation. Different lowercase letters in a row indicate that the differences are statistically significant (P < 0.05). Observed species: Community richness; Shannon index: Community diversity; Simpson index: Community diversity; Chao1 index: Community richness. (DOCX) [file pone.0257432.s002.docx]

**S1 Table. Comparison of species numbers and 16S (bacterial) diversity indices observed in the different cropping systems**

| Cropping  system | Observed species | Shannon index | Simpson index | Chao1 index |
| --- | --- | --- | --- | --- |
| CK | 2839.33 ± 112.34b | 10.40 ± 0.07a | 1.00 ± 0.00a | 5129.79 ± 364.28a |
| Q | 2978.67 ± 25.12ab | 10.46 ± 0.02a | 1.00 ± 0.00a | 5472.59 ± 223.76a |
| B | 3242.33 ± 122.83a | 10.51 ± 0.09a | 1.00 ± 0.00a | 6031.56 ± 339.80a |
| D | 3051 ± 133.63ab | 10.58 ± 0.05a | 1.00 ± 0.00a | 5373.15 ± 449.44a |

CK: continuous tomato cropping; Q: celery/tomato rotation; B: cabbage/tomato rotation; D: kidney bean/tomato rotation. Different lowercase letters in a row indicate that the differences are statistically significant (P < 0.05). Observed species: community richness; Shannon index: community diversity; Simpson index: community diversity; Chao1 index: community richness.
