# Supplementary material for: Effects of different vegetable rotations on the rhizosphere bacterial community and tomato growth in a continuous tomato cropping substrate
Source: PLoS One. 2021 Sep 23;16(9):e0257432. doi: 10.1371/journal.pone.0257432 (PMC8459948; doi:10.1371/journal.pone.0257432)
Supplement: S2 Table — CK: Continuous tomato cropping; Q: Celery/tomato rotation; B: Cabbage/tomato rotation; D: Kidney bean/tomato rotation; Ci: Intercellular CO2 concentration; Tr: Transpiration rate; Gs: Stomatal conductance; Pn: Net photosynthetic rate. Different lowercase letters at each phenological stage indicate that the differences are statistically significant (P < 0.05). (DOCX) [file pone.0257432.s003.docx]

**S3 Table. Effects of different vegetable rotations on light and photosynthetic parameters of the tomato leaves**

| Cropping system | Ci | Tr | Gs | Pn |
| --- | --- | --- | --- | --- |
|  | (μmol·mol^-1^) | (mmol·m^-2^·s^-1^) | (mol·m^-2^·s^-1^) | (μmol·m^-2^·s^-1^) |
| CK | 377.5 ± 8.53b | 4.08 ± 0.28b | 322 ± 29.17b | 5.53 ± 0.44b |
| Q | 421.5 ± 11.42a | 6.65 ± 0.36a | 531.5 ± 19.4a | 8.21 ± 0.59a |
| B | 381.5 ± 4.84b | 6.9 ± 0.59a | 485.75 ± 18.87a | 7.25 ± 0.55ab |
| D | 398.5 ± 4.52ab | 6.4 ± 0.15a | 524.25 ± 24.85a | 6.1 ± 0.66b |

CK: continuous tomato cropping; Q: celery/tomato rotation; B: cabbage/tomato rotation; D: kidney bean/tomato rotation; Ci: intercellular CO_2_ concentration; Tr: transpiration rate; Gs: stomatal conductance; Pn: net photosynthetic rate. Different lowercase letters at each phenological stage indicate that the differences are statistically significant (P < 0.05).
