# Supplementary material for: Effects of different vegetable rotations on the rhizosphere bacterial community and tomato growth in a continuous tomato cropping substrate
Source: PLoS One. 2021 Sep 23;16(9):e0257432. doi: 10.1371/journal.pone.0257432 (PMC8459948; doi:10.1371/journal.pone.0257432)
Supplement: S3 Table — CK: Continuous tomato cropping; Q: Celery/tomato rotation; B: Cabbage/tomato rotation; D: Kidney bean/tomato rotation; Fv: Variable fluorescence; Fm: Maximum fluorescence; Fo: Initial fluorescence; qp: Level of photochemical quenching of photosystem II; NPQ: Non-photochemical quenching. Different lowercase letters at each phenological stage indicate that the differences are statistically significant (P < 0.05). (DOCX) [file pone.0257432.s004.docx]

**S4 Table. Effects of different vegetable rotations on the fluorescence parameters of tomato leaves**

| Cropping system | Fv/Fm | Fv/Fo | q^p^ | NPQ |
| --- | --- | --- | --- | --- |
| CK | 0.79 ± 0.01a | 3.28 ± 0.19a | 0.75 ± 0.01b | 0.23 ± 0.02a |
| Q | 0.82 ± 0.02a | 3.81 ± 0.29a | 0.76 ± 0.02ab | 0.12 ± 0.01b |
| B | 0.81 ± 0.01a | 3.72 ± 0.47a | 0.77 ± 0.01ab | 0.13 ± 0.01b |
| D | 0.8 ± 0.01a | 3.42 ± 0.3a | 0.78 ± 0.003a | 0.16 ± 0.02ab |

CK: continuous tomato cropping; Q: celery/tomato rotation; B: cabbage/tomato rotation; D: kidney bean/tomato rotation; Fv: variable fluorescence; Fm: maximum fluorescence; Fo: initial fluorescence; q^p^: level of photochemical quenching of photosystem II; NPQ: non-photochemical quenching. Different lowercase letters at each phenological stage indicate that the differences are statistically significant (P < 0.05).
