# Supplementary material for: Effects of different vegetable rotations on the rhizosphere bacterial community and tomato growth in a continuous tomato cropping substrate
Source: PLoS One. 2021 Sep 23;16(9):e0257432. doi: 10.1371/journal.pone.0257432 (PMC8459948; doi:10.1371/journal.pone.0257432)
Supplement: Attachment — Submitted filename: Response to Reviewer 2.docx [file pone.0257432.s005.docx]

**Response to Comments of Reviewer # 2**

Q1. *My main observation is about the result shown in Table 1.*

*In Results section, I disagree with the authors, according to my opinion all these indices (Shannon, Simpson, Chao1 index), differences were not significant, however, an increased number of observed species occurred in B (cabbage/tomato) compared to the CK (continuous tomato cropping). I consider that this paragraph should be rewritten and then modified in the discussion.*

*According to my experience, when the differences are not significant, I only can say that there is a trend and cannot order from highest to lowest.*

**Response:** Thank you for your kind comment. For table 1, we have made corresponding modifications in the results and discussion. The corresponding sentences in the manuscript are as follows:

**Result：**“Additionally, compared with CK, the 3 vegetable rotations showed an increasing trend for the observed species 、Shannon index and Chao1 index of bacteria.”(Line180-181)

**Discussion:** “In our study, bacterial alpha-diversity analysis revealed that the observed species of the B (cabbage) rotation were the highest, which was significantly higher than CK; that is, the B rotation had the highest bacterial richness, followed by the Q (celery) rotation. Meanwhile, compared with CK, observed species, Shannon index and Chao1 index of all the three treatments including Q, B and D showed an increasing trend, indicating that crop rotation could improve the abundance and diversity of substrate bacteria. Therefore, compared with continuous monoculture, rotation can improve the diversity and richness of soil bacterial communities, as has been reported in many studies.” (Line303-310)

Q2. *In Fig. 2, I suggest* *increasing the letter size and put it in horizontal form.*

**Response:** Thank you for your kind suggestion. We have increased the letter size in Figure 2 and put it in horizontal form. The modified Figure 2 is as follows:

Q3. *In Fig.4, I recommend deleting g_ in each bacteria name to help to understand the analysis.*

**Response:** Thank you for your kind comment. After our consideration, we decided to delete Fig. 4 from the manuscript. Because reviewer #1 suggested deleting Fig. 4. We also consulted the editor again, and the editor also thought that Fig. 4 was of little significance. In addition, the deletion of Fig. 4 does not affect the conclusion of the full text. Therefore, we deleted Fig. 4 from the manuscript. Thank you again for your valuable comments.

Q4. *I suggest moving the percentage date. For Example, on lines 279-80*

*“At the beginning of flowering, the height of the Q plant was the highest, being significantly higher than that of the CK plant (by 0.44%)”.*

*Please, change by “At the beginning of flowering, the height of the Q plant was the highest (0.44%), being significantly higher than that of the CK plant.*

**Response:** Thank you for your helpful suggestion. We have changed by “At the beginning of flowering, the height of the Q plant was the highest (0.44%), being significantly higher than that of the CK plant.” (Line250)

Q5. *On lines 281-282: “At the early fruiting stage, the D plant was significantly taller (0.46%), than the CK plant. And so on with the next…*

*The results show that* *these growth parameters are slightly modified. Please consider it in the discussion section.*

**Response:** Thank you for your helpful suggestion. We have moved all the percentage date in the manuscript to the appropriate position. (Line252,261). In the discussion part, we further discussed the result of " these growth parameters are slightly modified ", and the corresponding sentences are as follows: “It was found that the height, stem diameter, and leaf area of subsequent tomato plants were basically the same throughout the growth period, and the changes among the different crop rotations were not significant. This may be because our rotation experiment has only been carried out for one year, so the effect on tomato growth is not obvious.” (Line341-344)

Q6. *In the M&M section, I consider that the sentence “The materials and methods are similar to those of “Lyu” [2]:” maybe incorporated inside experimental design.*

**Response:** Thank you for your kind comment. We have deleted " The materials and methods are similar to those of “Lyu” [2]: " in materials and methods and added " The Experimental design is similar to those of “Lyu” [2]: " in experimental design. (Line70)
